# Aluminum Parts Fabricated by Laser-Foil-Printing Additive Manufacturing: Processing, Microstructure, and Mechanical Properties

**DOI:** 10.3390/ma13020414

**Published:** 2020-01-16

**Authors:** Chia-Hung Hung, Yingqi Li, Austin Sutton, Wei-Ting Chen, Xiangtao Gong, Heng Pan, Hai-Lung Tsai, Ming C. Leu

**Affiliations:** 1 Department of Mechanical and Aerospace Engineering, Missouri University of Science and Technology, Rolla, MO 65409, USA; yl534@mst.edu (Y.L.); atsfk6@mst.edu (A.S.); xgxfv@mst.edu (X.G.); hp5c7@mst.edu (H.P.); tsai@mst.edu (H.-L.T.); mleu@mst.edu (M.C.L.); 2 Materials Research Center, Missouri University of Science and Technology, Rolla, MO 65409, USA; chenweit@mst.edu

**Keywords:** additive manufacturing, laser-foil-printing, aluminum alloys

## Abstract

Fabrication of dense aluminum (Al-1100) parts (>99.3% of relative density) by our recently developed laser-foil-printing (LFP) additive manufacturing method was investigated as described in this paper. This was achieved by using a laser energy density of 7.0 MW/cm^2^ to stabilize the melt pool formation and create sufficient penetration depth with 300 μm thickness foil. The highest yield strength (YS) and ultimate tensile strength (UTS) in the LFP-fabricated samples reached 111 ± 8 MPa and 128 ± 3 MPa, respectively, along the laser scanning direction. These samples exhibited greater tensile strength but less ductility compared to annealed Al-1100 samples. Fractographic analysis showed elongated gas pores in the tensile test samples. Strong crystallographic texturing along the solidification direction and dense subgrain boundaries in the LFP-fabricated samples were observed by using the electron backscattered diffraction (EBSD) technique.

## 1. Introduction

The additive manufacturing (AM) process is commonly used to create complex-shaped three-dimensional objects that are difficult to fabricate by traditional machining processes [1]. The flexibility of the AM process has allowed many different materials (e.g., Ti-6Al-4V, 304L, 316L, IN718, and aluminum alloys) to be fabricated [2]. Aluminum alloys have been widely used in aerospace, automotive, and structural applications that require materials having a high strength-to-weight ratio, thermal conductivity, and corrosion resistance [3,4]. Although using the AM technology to create complex-geometry parts of aluminum alloys is attractive, not much research has been conducted [5,6] on aluminum alloys fabricated by AM processes with the exception of AlSi10Mg [7,8,9,10,11]. The addition of silicon in aluminum alloys is done to reduce oxygen absorption and enhance melt pool fluidity. This reduces the oxidation and increases the wettability between successive layers. Silicon has also been shown to increase the powder flowability in powder-bed fusion based AM processes [7,12]. AM of aluminum alloys with low silicon content has not been extensively studied because of processing difficulty. AM of aluminum is exacerbated in powder-bed fusion processes due to the high percentage of volumetric reduction and the poor fluidity of molten metal associated with aluminum powder during melting and solidification, leading to the high porosity (~10% porosity) of those AM-fabricated part [13]. Moreover, the surface area of the powder promotes more oxidation, which could be detrimental to the part properties [13,14]. Therefore, processing aluminum powder to create fully dense parts has been proven difficult to achieve.

The study reported in the present paper used a different approach known as the laser-foil-printing (LFP) process, which utilizes foil as the feedstock to alleviate the problems associated with powder processing described above. Laser-foil-printing is a laminated object manufacturing process developed at the Missouri University of Science and Technology. It has been used to build 3D-structural parts of Zirconium-based amorphous metals [15] and crystalline metals such as carbon steel and stainless steel [16,17] layer by layer. LFP uses a dual-laser system to weld each layer of metal foil onto the substrate or a previously fabricated layer and then cut the cross-sectional contour for the newly created layer. In the LFP process, the thickness of foil can be tens of micrometers to hundreds of micrometers. The cooling rate of melt pool using the metal foil as the feedstock is high enough to generate fine crystalline grain structures or even amorphous structures if desired [15,17] because the thermal heat of the melt pool can be conducted away efficiently through the foil, instead of powder whose thermal conductivity is significantly lower than the foil [18]. Furthermore, the formation of shrinkage pores can be minimized because the usage of foil does not involve high volumetric reduction during the melting and solidification process.

In this study, the laser welding process window of Al-1100 was investigated and optimized through cross-sectioning of single-layer samples with various process parameters. Utilizing the optimized parameters, Al-1100 parts were fabricated by the LFP process. The fabricated samples were investigated by quantifying their mechanical properties through tensile tests and visualization of microstructure using metallography and electron back-scattered diffraction, in comparison to annealed aluminum samples [5,6].

## 2. Process Overview and Experimental Setup

### 2.1. Laser Foil Printing (LFP)

**Figure 1:**
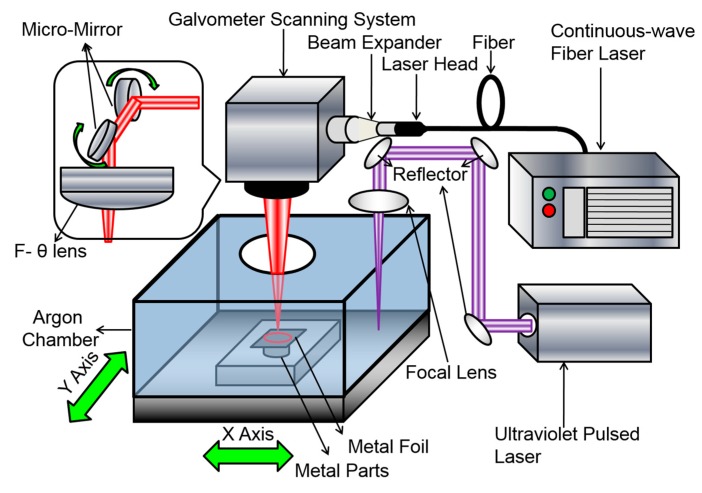
Schematic illustration of the LFP system.

Al-1100 (commercial grade pure aluminum, >99% aluminum) foil is used as the feedstock in this study. The thickness of the foil was 150 μm, which is readily available commercially. The LFP system consists of a continuous-wave (CW) fiber laser (IPG YLP-1000, NY, USA) for welding and an ultraviolet (UV) pulsed laser for cutting, as schematically shown in Figure 1. The CW fiber laser subsystem includes a galvo-mirror scanner (SCANLAB) and an F-θ lens. The UV pulsed laser (Coherent AVIA-355X, CA, USA) subsystem includes optical reflection mirrors, a focal lens, and high-precision Aerotech motor-driven stages. The CW fiber laser has a center wavelength of 1070 nm, beam quality factor M^2^ of 3.04, and maximum average power output of 1000 W. The focal length of the F-θ lens is 330 mm, and the laser spot size (d) is ~160 μm. For the UV laser, its center wavelength, pulse width, and maximum average power output are 355 nm, 30 ns, and 10 W, respectively. The focus length of the lens is 100 mm and the laser spot size is 40 μm. Both the CW and UV laser beams are focused on the foil surface.

**Figure 2:**
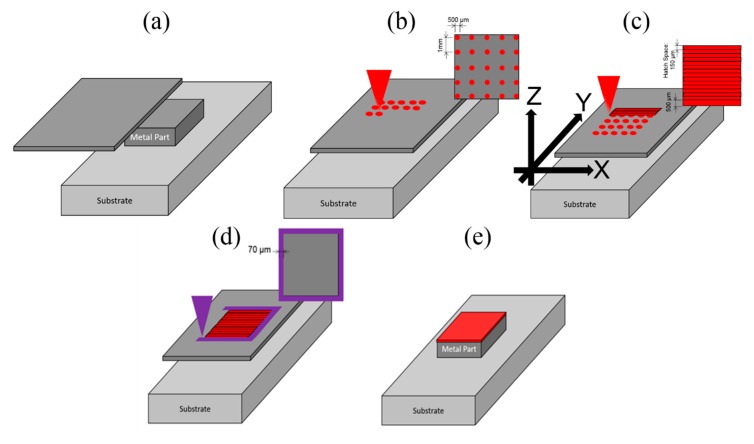
Schematic illustrations of the five steps in laser-foil-printing for the processing of each layer: (**a**) foil feeding; (**b**) spot welding; (**c**) pattern welding; (**d**) contour cutting; (**e**) excess foil removing.

To build an aluminum part using the LFP process, five steps are followed for each layer, as illustrated in Figure 2. First, a layer of metal foil is placed onto the substrate or a previously welded layer (see Figure 2a). Next, spot welding is applied on the metal foil using the CW fiber laser (see Figure 2b). The purpose of spot welding is to fix the foil onto the previous layer to prevent the foil from possible thermal distortion/curving. The third step is pattern welding which uses a meander scan strategy, as shown in Figure 2c. The foils were welded under an argon shielding atmosphere with an oxygen content of ~1%. The argon shielding gas flows into the chamber through the bottom of the chamber and flows out of the chamber through the open window on the top side of the chamber as shown in Figure 1. The fourth step is to cut the pattern’s contour using the UV pulsed laser (see Figure 2d). Finally, the excess foil is removed (see Figure 2e). Note that the foils can be pre-cut into the shape for each layer according to the CAD file of the part and then the cut foils are welded together layer by layer. In Figure 2c, the *x* axis is parallel to the laser scanning direction on the layer plane; the *y* axis is perpendicular to the laser scanning direction on the layer plane; the *z* axis is parallel to the layer building direction.

In this study, Al-1100 is selected as the material in the LFP process since it has the highest thermal conductivity, highest optical reflectivity, highest oxidation tendency, high thermal expansion, and low percentage of volatile elements among the aluminum alloys that are considered not suitable for the powder-bed fusion processes, which is very challenging because of the increase in oxidation layer formation and the difficulty of stabilizing the melt pool formation [13,14]. While the Al-1100 powder has unfavorable physical properties, using foil as the feedstock is ideal for Al-1100 because it circumvents those aforementioned issues by minimizing the surface exposure, oxidation, and pore formation. A 6-mm-thick Al-1100 plate was used as the substrate. To increase the fabrication efficiency, two foils (thickness of 150 µm for each foil) were stacked and welded together at the same time using a single weld, which means a thickness (*s*_1_) of 300 µm for every layer. For spot welding, the laser power was 700 W, the weld time per spot was 7 ms, and the distance between spots was 1 mm. For cutting the pattern’s contour, the pulse energy was 0.16 mJ with the pulse repetition rate of 4 kHz and the cutting speed of 1 mm/s. For pattern welding, the process parameters were investigated using various laser powers and speeds with the hatch space (*h*) of 0.15 mm. The laser power (*P*) ranged from 630 to 700 W, the laser scan speed (*v*) was 100–400 mm/s, and the spot size (d) was 160 µm. The volumetric energy input (VEI) and the power density (P_d_) of laser pattern welding can be calculated using the equations of *VEI* = *P*/(*v*·*h*·*s*_1_) and *P_d_* = 8*P*/(*πd*^2^), respectively [19].

**Figure 3:**
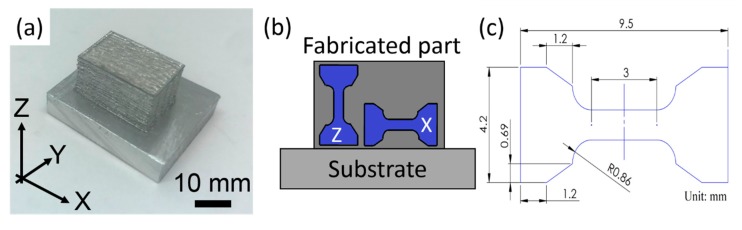
(**a**) Al-1100 alloys fabricated by LFP; (**b**) side view of a part fabricated by LFP showing how the *x* axis direction and *z* axis direction tensile specimens were extracted; (**c**) dimensions of the tensile specimen.

**Figure 4:**
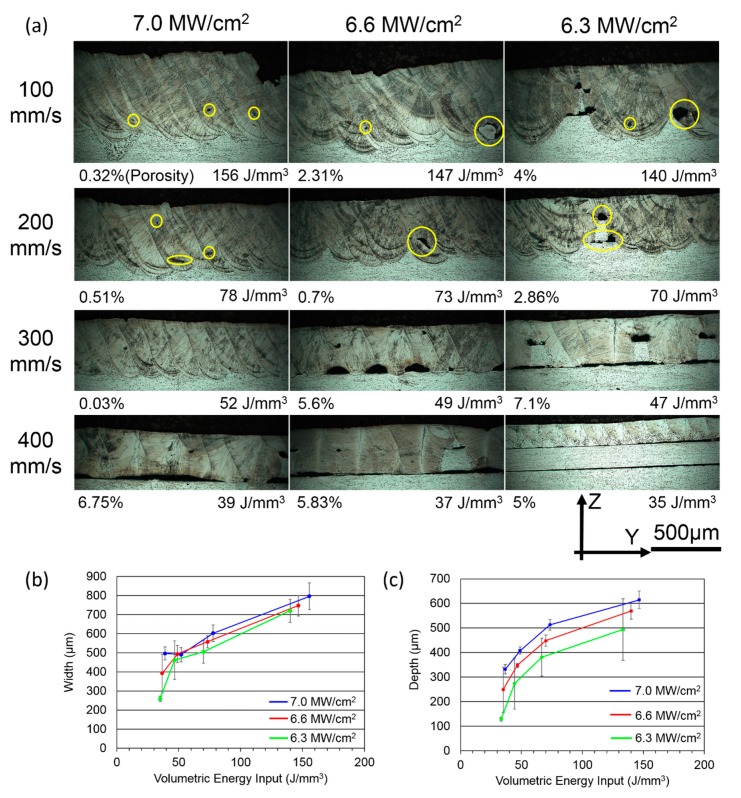
(**a**) Cross-sectioned OM images in Y-Z plane at different levels of power density (MW/cm^2^), volumetric energy input (J/mm^3^), and scanning speed (mm/s); the porosity of each image is given at its bottom left; (**b**) width of melt pool; and (**c**) depth of melt pool vs. VEI at the different levels of power density: 6.3 MW/cm^2^ (Green), 6.6 MW/cm^2^ (red), and 7.0 MW/cm^2^ (blue).

To build an LFP-fabricated Al-1100 block with the dimensions of 18 mm (length) × 12 mm (width) × 10 mm (height), as shown in Figure 3, the optimal process parameters, with which the melt pools are penetrated to the substrate and stably formed with the lowest porosity, were chosen from Figure 4a through a parametric study. In Figure 3a, the *x* axis is parallel to the laser scanning direction; the *y* axis is perpendicular to the laser scanning direction; the *z* axis is parallel to the layer building direction.

### 2.2. Characterization

The LFP-fabricated aluminum blocks were then cut off from the substrate, using the wire electrical discharge machining (WEDM, Sodick VZ300L Wire EDM machine, Warwickshire, UK), for analysis. The oxygen determinator (Leco TC500, MI, USA) was used to measure the oxygen content using the carrier-gas hot extraction method. The microstructure and the fracture surface were characterized by using an optical microscope (OM, Nikon Epiphot 200, Tokyo, Japan), an X-ray diffraction instrument (XRD, Philips X’pert MRD, Amsterdam, Netherlands), and a scanning electron microscope (SEM, Helios Nanolab 600, OR, USA) equipped with electron backscattered diffraction (EBSD) and energy-dispersive X-ray spectroscopy (EDS) detectors. The specimens for OM and EBSD analyses were polished utilizing standard metallographic techniques using #320 grind paper, with 9 µm, 3 µm, or 1 µm diamond suspensions, and 0.04 µm silica. The specimens were etched by immersion in Keller’s Reagent (95% deionized water, 2.5% nitric acid, 1.5% hydrochloric acid, and 1% hydrogen fluoride) for revealing the melt pools. The porosity was determined by calculating the total area of the pores in OM images of the cross-section with reference to the total area of 63 mm^2^. The area of each pore was measured using ImageJ software (Version 1.5) [8]. The EBSD patterns had the scanning area of 600 × 600 μm^2^ with a step size of 2 μm. The average grain size in each EBSD pattern was calculated by following the ASTM E2627-13 standard [20].

The tensile strengths of the LFP-fabricated Al-1100 specimens were measured along the layer building direction (indicated by “Z”) and along the laser scanning direction (indicated by “X”). The tensile specimens as shown in Figure 3b were 2 mm thick, and they were cut from the fabricated aluminum blocks using WEDM in order to avoid the surface effect on the mechanical properties [21]. The tensile tests were conducted on an INSTRON machine with a clip-on extensometer at room temperature at the machine crosshead speed of 0.015 mm/mm/min (strain rate per minute). Five LFP-fabricated tensile specimens in both X and Z directions and the annealed aluminum parts were tested, and the mean value with the standard deviation of each test was reported. The annealed aluminum parts were fabricated by following the ASTM-B209 standard with annealing heat treatment [22]. One-way ANOVA analysis was used to analyze the tensile test data.

## 3. Results and Discussion

In order to ascertain the feasible process parameters for building Al-1100 parts using the LFP process, we conducted a parametric study by investigating the effect of depth and width of melt pool with various laser power densities, volumetric energy inputs (VEIs), and laser scanning speeds on Al-1100 alloys. The porosity of each image in Figure 4a was measured and shown at the bottom left of each image. We also examined the specimens’ cross-sections, which are shown in Figure 4. The width and depth of a melt pool in Figure 4a at different levels of VEI, power density, and scanning speed were measured and summarized in Figure 4b,c, respectively, along with their standard deviations. Figure 4a shows that the power density of 6.3 MW/cm^2^ was not sufficient to create penetrating and stable melt pools even using the slowest scanning speed of 100 mm/s. Based on the large standard deviation of melt pool depth at the laser power density of 6.3 MW/cm^2^ in Figure 4c, the melt pool was unstable because the high thermal conductivity of Al-1100 could quickly conduct heat away, causing the situation of lacking laser energy. Therefore, the power density needed to increase in order to have stable melt pools. As the power increased to 6.6 MW/cm^2^, the melt pools were stably formed at the VEI of 73 & 147 J/mm^3^; however, pores were found with the higher energy inputs and the top surface became rough. As the power density reached 7.0 MW/cm^2^, the formation of melt pools was stabilized at the minimum VEI of 52 J/mm^3^. However, as the VEI increased to 78 J/mm^3^, micro-pores (marked by yellow circles in Figure 4a) were observed at the bottom of the melt pool, which is a typical feature in the keyhole mode of laser welding [23]. In Figure 4c, the standard deviations of melt pool width and depth at 7.0 MW/cm^2^ were relatively small in comparison to the other VEIs because the laser power density was high enough to provide sufficient heat flux for overcoming the high thermal conduction mechanism and stabilizing the formation of melt pool. With the increase in power density, Marangoni flow of the melt pool could change from a negative surface tension gradient (the molten metal flows from the center toward the edge of the melt pool and causes a small penetration depth) to a positive surface tension gradient (the molten metal flows from the edge toward the center of the melt pool and causes a larger penetration depth) [24]. Therefore, the threshold of power density for producing a stable melt pool was identified as 7.0 MW/cm^2^. This is the highest threshold of power density reported in the literature among aluminum alloys because Al-1100 does not contain volatile elements (e.g., magnesium, zinc, lithium) that can help in stabilizing the formation of melt pool [25,26,27]. Hereafter, the desirable process parameters of 7.0 MW/cm^2^, 300 mm/s, and 52 J/mm^3^ were used to build Al-1100 parts using the LFP process.

The aluminum oxide film (thickness < 10 nm) could form on the surface of the melt pool [28]. It is well known this film has tenacious and strong physical properties. However, because of its nano-scale thickness, this film may be torn apart by the expansion of aluminum on melting, and the separated regions move aside by Marangoni flow or possibly by the evaporation of the aluminum suboxide (Al_2_O).

**Figure 5:**
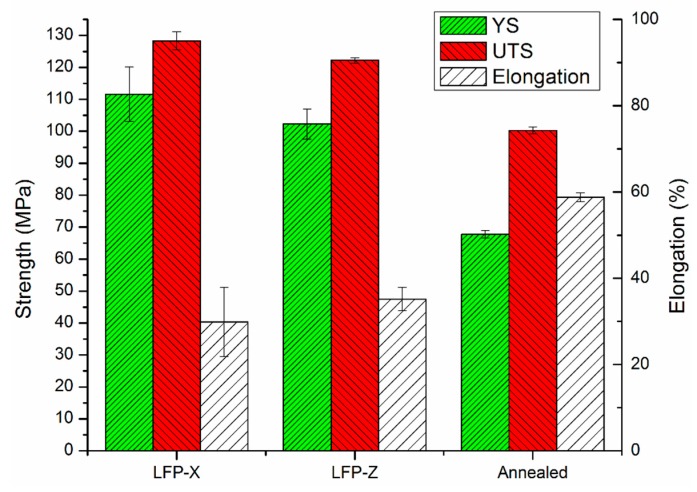
Tensile properties of the annealed and LFPed aluminum specimens in the laser scanning (LFP-X) and part building (LFP-Z) directions.

**Figure 6:**
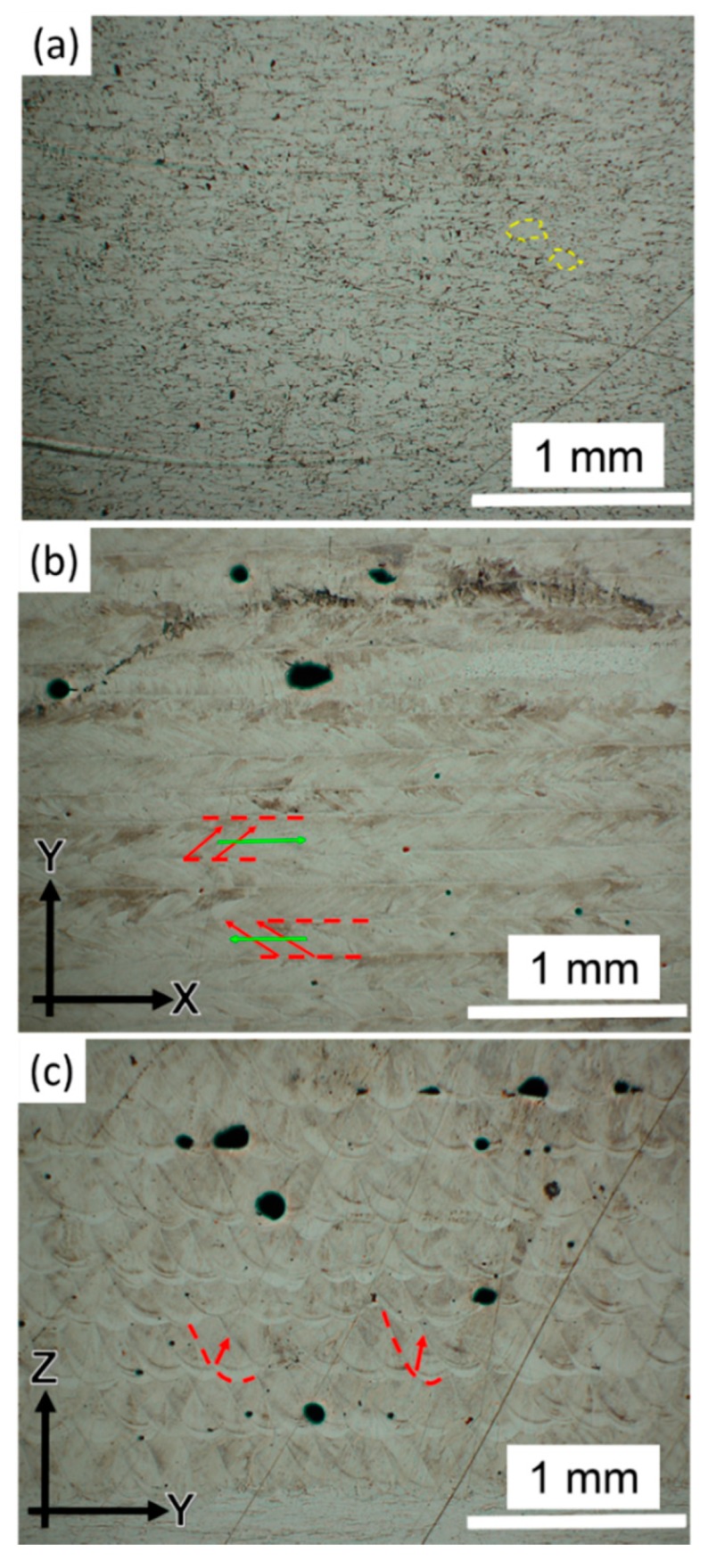
OM images of the etched surface of (**a**) annealed aluminum part; (**b**) LFP-fabricated part in X-Y plane; (**c**) LFP-fabricated part in Y-Z plane. Yellow dashed lines in (**a**) represent the grain boundary; red dashed lines in (**b**) and (**c**) represent the boundry of melt pool; the red arrows in (**b**) and (**c**) represent the grain growth direction; green arrows represent the laser scanning direction in (**b**).

The tensile properties of the annealed aluminum and LFP specimens were measured using standard tensile tests. Figure 5 presents the results of tensile testing on the annealed aluminum and the LFP-fabricated specimens in the laser scanning and layer building directions with the yield strength (YS), ultimate tensile strength (UTS), and the elongation at the breaking point with one standard deviation. It can be seen that the strength of the LFP parts is higher in both directions than the annealed parts. The YS and UTS of LFP-Z specimens are 51% and 22% higher, respectively, than the annealed specimen. However, the ductility of LFP-Z part is 40% less when compared to the annealed part; this is due to the existence of gas pores as shown in Figure 6b [29]. In Figure 5, the standard deviation of YS and elongation of the LFP-fabricated parts are larger than the annealed parts due to the presence of early failures in the annealed aluminum parts caused by porosity within the gauge section [29]. Moreover, Figure 5 reveals higher tensile strength along the laser scanning direction (LFP-X) than the part building direction (LFP-Z) but slightly lower elongation along the laser scanning direction than the part building direction. The ANOVA analysis was conducted to analyze the tensile test data on the difference between LFP and annealed specimens, with the result given in Table 1. The ANOVA result indicates that the differences between LFP and annealed specimens in YS, UTS, and strain are statistically significant.

Since tensile properties are known to be affected by oxygen [30], the oxygen contents were measured. The oxygen contents of the foil, annealed, and LFP-fabricated parts measured were 73 ± 21 ppm, 306 ± 31 ppm, and 372 ± 59 ppm, respectively. Although the oxygen content of the fabricated part increased by ~300 ppm after LFP process compared to the oxygen content of the foil, implying oxygen was absorbed by the molten metal during solidification in the build chamber. The oxygen contents between the annealed and LFP-fabricated parts are similar.

**Figure 7:**
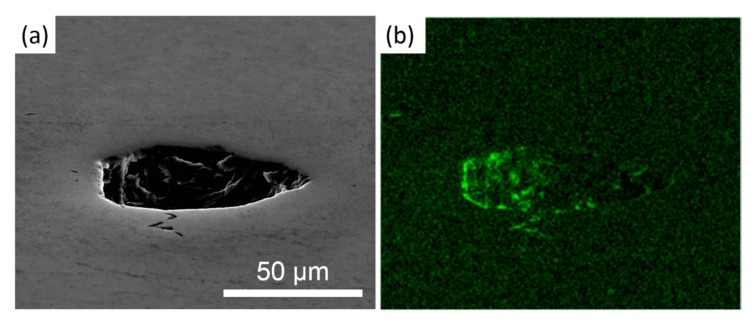
(**a**) SEM image of a pore of LFP-fabricated specimen and (**b**) its EDS mapping of oxygen content.

The annealed aluminum and LFP-fabricated parts were cross-sectioned and etched for revealing their microstructures and melt pool traces, as shown in Figure 6. The porosities of the annealed and LFP-fabricated parts were 0.1% and 0.7%, respectively. The geometries of pores within the LFP-fabricated part in Figure 6b,c are dome-shaped and globular. To measure the oxygen content of pores, one of the pores in the X-Y plane of a LFP-fabricated part was chosen to conduct the energy-dispersive X-ray spectroscopy (EDS) measurement. The SEM image of the pore is shown in Figure 7a. The oxygen content of the pore in Figure 7a is shown in Figure 7b, which indicates that high oxygen content was accumulated on the pore’s shell in Figure 7a due to abundant aluminum oxide formed during the melt solidification. The geometry and oxidation indicate that the voids were either pores generated from the bubbles of dissolved gas during the solidification of melt pool at the interface between the solidifying melt pool and the surrounding solid [31], or due to entrained gas in the gaps between the foil and the substrate [32]. Moreover, the etched surface of an annealed specimen in Figure 6a exhibits typical equiaxed grain boundaries (yellow dashed lines), while Figure 6b,c pertaining to the X-Y plane and Y-Z plane cross-sections of LFP processed parts, respectively, contain columnar grains indicative of a high cooling rate. In Figure 6b, the growth direction of columnar grains was observed to originate from the edge of a melt pool (red dashed lines) toward its center with an angle of ~45 degrees with respect to the laser beam scanning direction (green arrows). The columnar grains in Figure 6c grew from the boundary of a melt pool (red dashed line) toward the center line of melt pool as shown by red arrows. The chemical compositions of the LFP-fabricated and the annealed specimens were measured by the EDS technique, and the results are given in Table 2. High purity aluminum content (>99.3 wt. %) was measured in both specimens.

**Figure 8:**
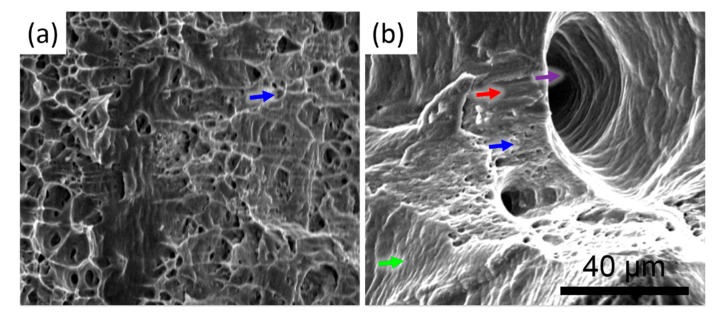
SEM images showing the fracture surface of the tensile aluminum specimens: (**a**) annealed; (**b**) LFP-X. The red, green, blue, and purple arrows represent quasi-cleavage, slip regime, dimple, and elongated pore, respectively.

The fracture surface of the annealed part, which has the highest ductility of the tensile specimen, is compared with the fracture surface of the LFP-X part, which has the lowest ductility of the tensile specimen, as shown in Figure 8. Fine, dense dimples were observed in the fracture surface of the annealed part as shown in Figure 8a. This is a typical fracture feature of a ductile material formed during the microvoid coalescence. However, since there were some gas pores formed during the LFP process, the fracture surface of the LFP part contains quasi-cleavages, slip regimes, sparse dimples, and elongated gas pores, as shown in Figure 8b [33,34]. Thus, the parts fabricated by the LFP process have less ductility.

EBSD analysis was performed on the LFP-fabricated and annealed samples to access the crystallographic texture and spatial distribution of the Al-1100 grains. The analyses were conducted on the 600 × 600 µm^2^ polished surfaces on the X-Y and Y-Z planes of the LFP-fabricated samples. All EBSD orientation maps and the pole figures acquired from the LFP-fabricated samples are presented with respect to the local growth direction to simplify the discussion. The grain microstructure of the annealed sample was also investigated by using EBSD. Note that only face-centered cubic (f.c.c.) crystal structure was observed in all EBSD data.

**Figure 9:**
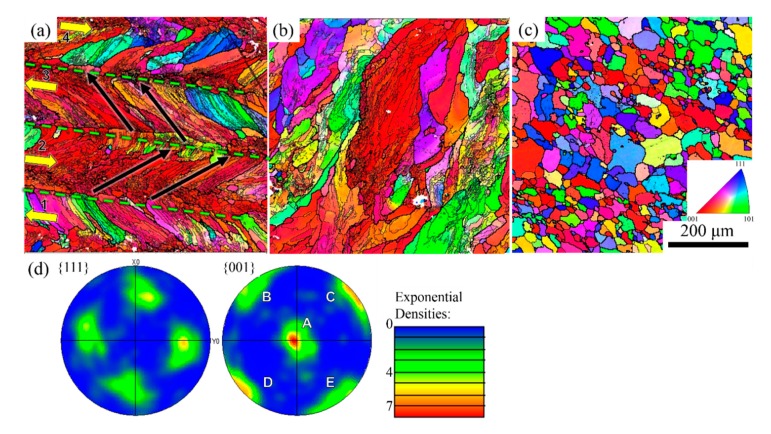
EBSD patterns of (**a**) X-Y plane of an LFP-fabricated aluminum part, (**b**) Y-Z plane of the LFP part, and (**c**) an annealed aluminum part. In (**a**), the laser scan direction, the grain growth direction, and the melt pool boundaries are marked by yellow arrow, black arrow, and green dashed line, respectively. Thicker boundary lines in (**a**)–(**c**) represent the high-angle grain boundaries (misorientation angle >15°, HAGB), while thinner boundary lines represent the low-angle grain boundaries (misorientation angle: 2° to 15°, LAGB). (**d**) The pole figures from {001} and {111} reflections were acquired from the area shown in (**a**).

As shown in Figure 9a, the representative EBSD orientation map reveals the spatial distribution and crystallographic orientations of Al-1100 grains with respect to the local grain growth direction on the X-Y plane of the LFP-fabricated sample. Columnar grain structure was observed, with an average grain size of 42.1 ± 10 µm. The growth of columnar grains followed a solidification path toward the heating source, hence the grains grew from the boundary of melt pool toward the center of melt pool. Figure 9a shows the grain growth direction at a ~45° tilt angle off the laser scanning track because of the temperature gradient of the melt pool. This observation is consistent with the microstructure shown in Figure 6b. A high density of low-angle grain boundary (LAGB, misorientation angle: 2° to 15°) was found in the columnar grains as shown in Figure 9a, with the subgrain size of 6 ± 3 µm, which is much smaller than the normal grain size. The variety of subgrain density was related to the local temperature gradient of the melt pool, because the cooling rate at the boundary of melt pool is higher than the centerline of melt pool and forms more subgrains at the boundary. The subgrains were observed on the Y-Z plane of LFP-fabricated sample as shown in Figure 9b. These naturally-formed LAGBs have also been reported in the parts fabricated by the selective laser melting process [35]. On the other hand, equiaxed grains with an average grain size 20.1 ± 3.5 µm were observed in the annealed sample as shown in Figure 9c. Few LAGBs were found in the annealed sample.

The relationships between grain/subgrain size and mechanical properties were also studied. As reported earlier (Figure 5), higher YS and UTS were measured in the LFP-fabricated sample compared to the annealed sample. This is attributed mainly to the formation of a large number of small subgrains during the solidification of melt pool in the LFP process. The subgrain boundaries inhibit dislocation movement when stress is applied, hence the mechanical strength of the material is enhanced. However, pileups of dislocations at the grain/subgrain boundaries in the LFP-fabricated sample result in a reduction in ductility (Figure 5).

The crystallographic texture of the LFP-fabricated sample on the X-Y plane is presented in the averaged pole figures shown in Figure 9d. The intensities in the {001} pole figure are dominated by the regions appearing in red in Figure 9a, indicating that Al-1100 columnar grains preferentially grew along this orientation. The other intensity clusters in the {001} pole figure, labeled B, C, D and E in Figure 9d, are associated with symmetrically equivalent poles in the f.c.c. structure, which are inclined by 90°. As expected, the intensity clusters in the {111} pole figure observed are 45°-tilt away from the {001} poles. The mosaicity in the growth direction was measured ~18° from the maximum to minimum of the intensity cluster labeled in A, indicating variation of grain orientation.

## 4. Conclusions

Additive manufactured parts of Al-1100 aluminum alloy by the laser-foil-printing (LFP) process have been investigated. The process parameter window was studied through a combination of different levels of power density, scanning speed, and volumetric energy input to find the threshold of power density for stabilizing the melt pool formation during the laser welding process. Al-1100 specimens were built and a relative density of 99.3% was achieved using the LFP process. The yield strength, ultimate tensile strength, and ductility of the Al-1100 specimens fabricated by this additive manufacturing process were measured and compared to the annealed aluminum parts. The yield strength and ultimate tensile strength of the LFP specimens were 51% and 22% higher, respectively, but the ductility was 40% lower. Electron backscattered diffraction patterns showed low-angle-grain-boundary subgrains formed within high-angle-grain-boundary grains due to the fast cooling of the LFP process, with the dominant grain growth orientation of {001}.

## Figures and Tables

**Table 1:** ANOVA analysis of tensile test data for LFP and annealed specimens.

Mechanical Property	Source	DOF	Adj SS	F-Value	*p*-Value
YS	Process	1	5114.8	112.16	0.000
	Error	13	592.8		
UTS	Process	1	2076.3	204.56	0.000
	Error	13	132.0		
Strain	Process	1	2304.7	82.89	0.000
	Error	13	286.5		

**Table 2:** The chemical compositions (wt. %) of LFP-fabricated and annealed specimens.

Element	Fe	Mn	Cu	Si	Zn	Al
LFP	0.4	0.0	0.0	0.2	0.0	Bal.
Annealed	0.5	0	0.2	0	0	Bal.
